# Supplementary material for: Nr6a1 controls Hox expression dynamics and is a master regulator of vertebrate trunk development
Source: Nat Commun. 2022 Dec 15;13:7766. doi: 10.1038/s41467-022-35303-4 (PMC9755267; doi:10.1038/s41467-022-35303-4)
Supplement: Supplementary file 1 — Supplementary Information [file 41467_2022_35303_MOESM1_ESM.pdf]

Supplementary Information File

Supplementary Figures 1-13

Supplementary Table 1

Supplementary Figures

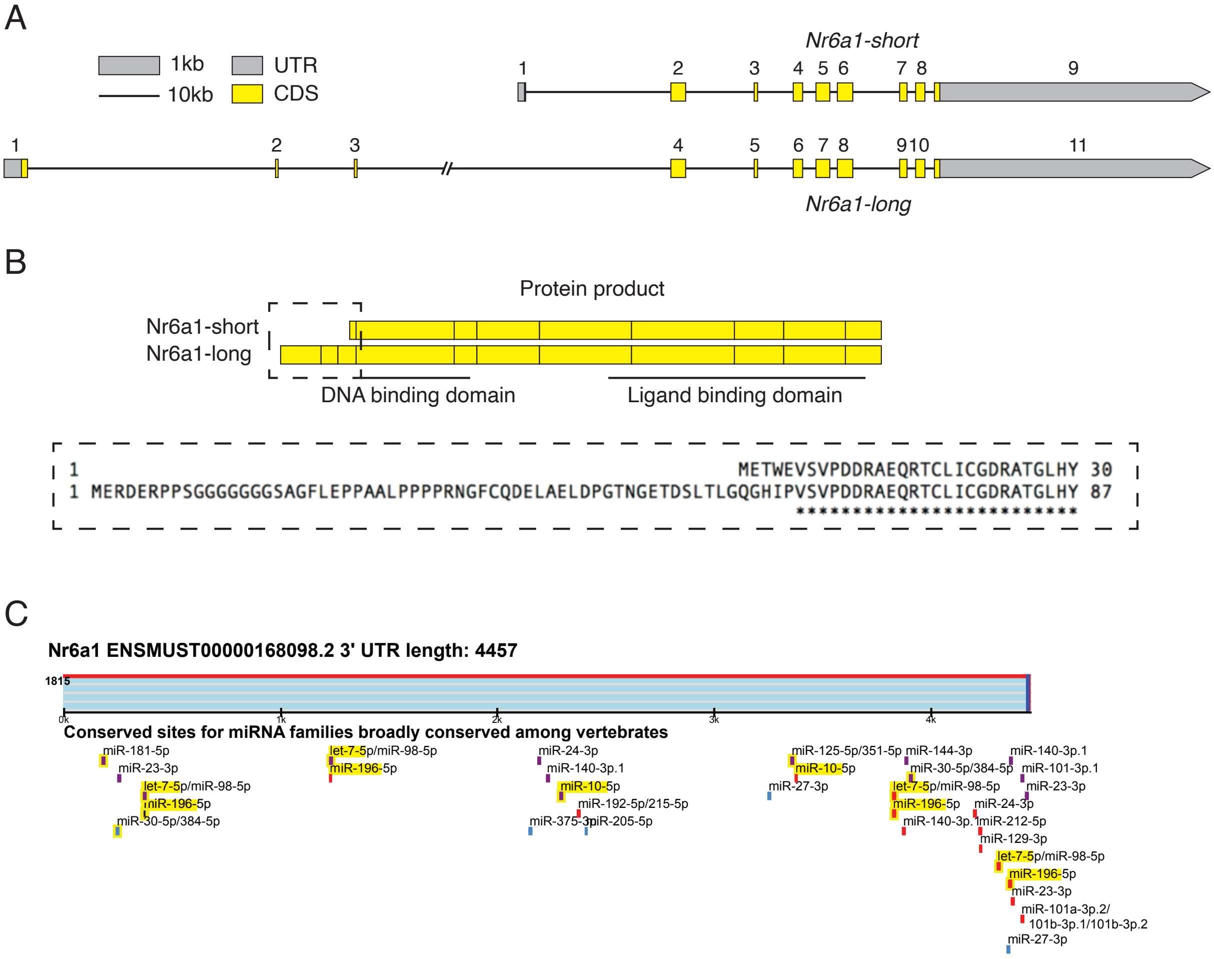

Supplementary Figure 1. The *Nr6a1* genomic locus generates multiple isoforms in the mouse.

A. The genomic structure of *M.musculus Nr6a1* short and long isoforms, drawn approximately to scale, indicating the position of exons (boxes) and introns (lines). Within exons, yellow represents coding sequence (CDS) and grey represents untranslated region (UTR).

B. The protein products (hatched yellow boxes) encoded by *Nr6a1* short and long isoforms share the majority of the DNA binding domain and the entirety of the ligand binding domain. Alternative start sites for each isoform produce variation in the protein sequence of the N-terminus as shown.

C. MicroRNA binding sites located within the *Mus musculus Nr6a1* 3' UTR, identified using TargetScan[40]. The binding sites of two *Hox*-embedded microRNAs, *miR-10* and *miR-196* were highlighted in yellow.

A

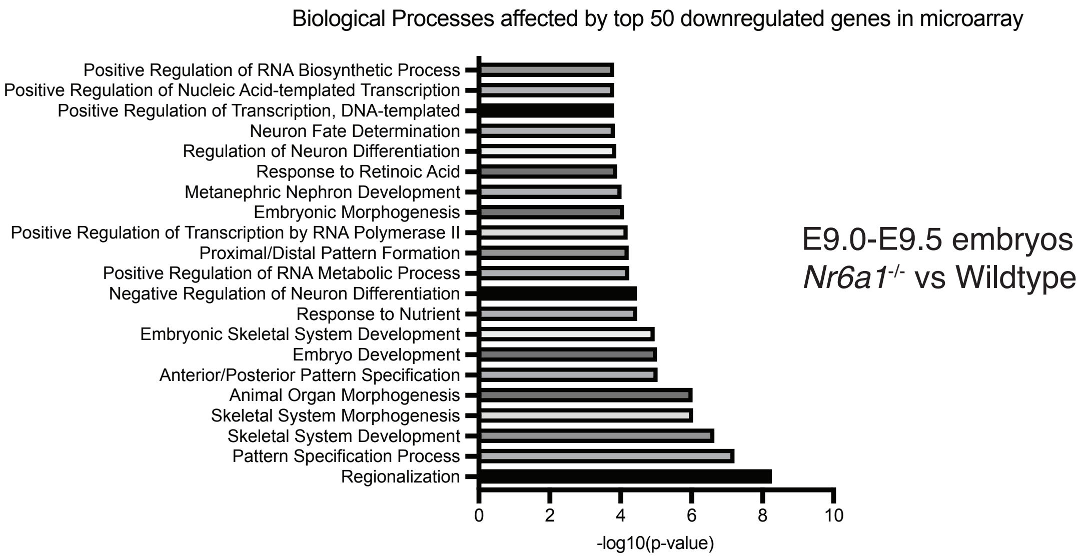

Biological Processes affected by top 50 downregulated genes in microarray
Positive Regulation of RNA Biosynthetic Process
Positive Regulation of Nucleic Acid-templated Transcription
Positive Regulation of Transcription, DNA-templated
Neuron Fate Determination
Regulation of Neuron Differentiation
Response to Retinoic Acid
Metanephric Nephron Development
Embryonic Morphogenesis
Positive Regulation of Transcription by RNA Polymerase II
Proximal/Distal Pattern Formation
Positive Regulation of RNA Metabolic Process
Negative Regulation of Neuron Differentiation
Response to Nutrient
Embryonic Skeletal System Development
Embryo Development
Anterior/Posterior Pattern Specification
Animal Organ Morphogenesis
Skeletal System Morphogenesis
Skeletal System Development
Pattern Specification Process
Regionalization
0 2 4 6 8 10
-log10(p-value)
E9.0-E9.5 embryos
Nr6a1-/- vs Wildtype

B

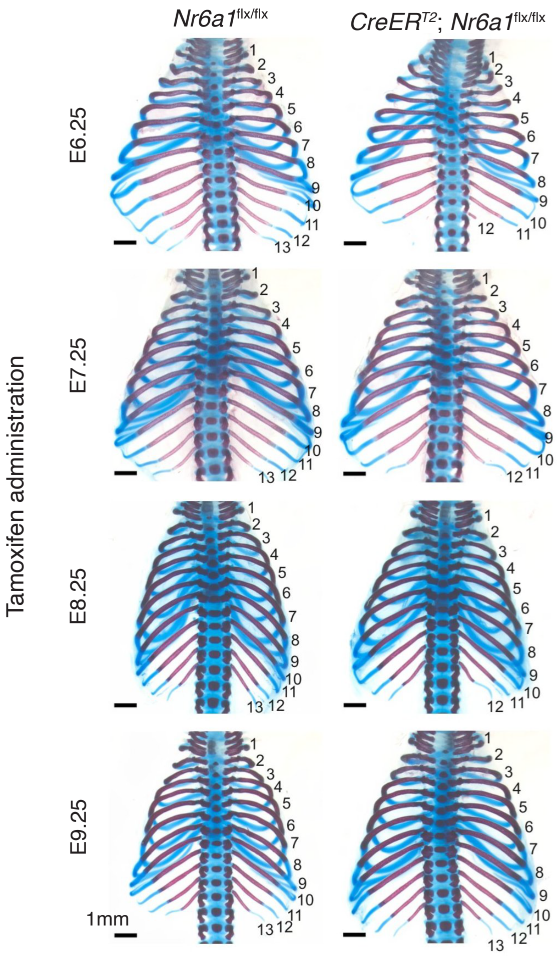

Nr6a1flx/flx
CreERT2; Nr6a1flx/flx
Tamoxifen administration
E6.25
E7.25
E8.25
E9.25
1mm

C

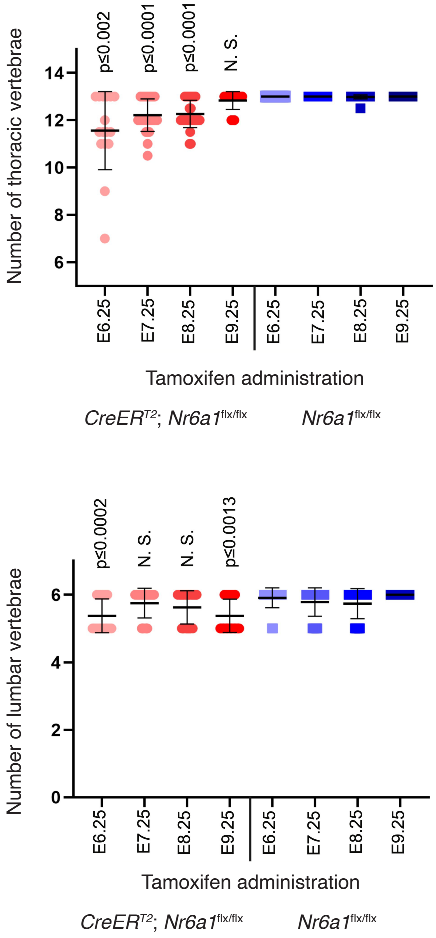

Number of thoracic vertebrae
p≤0.002
p≤0.0001
p≤0.0001
N. S.
E6.25 E7.25 E8.25 E9.25 E6.25 E7.25 E8.25 E9.25
Tamoxifen administration
CreERT2; Nr6a1flx/flx
Nr6a1flx/flx
Number of lumbar vertebrae
p≤0.0002
N. S.
N. S.
p≤0.0013

Supplementary Figure 2. Nr6a1 is required prior to E9.25 to control thoracic vertebral number

A. The predicted key biological processes disrupted in *Nr6a1*$^{-/-}$ mutants compared to wildtype controls, which was analysed by ToppGene[102] using the top 50 down-regulated genes in *Nr6a1*$^{-/-}$ mutants.
B. Characterisation of the ubiquitous but temporally-controlled deletion of *Nr6a1*, achieved by crossing *Cre*^{ERT2} and *Nr6a1*$^{flx/flx}$ lines and Tamoxifen (Tam) administration to pregnant dams on a single day of development (E6.25-E9.25) as indicated. E=embryonic day. Skeletal preparation of E18.5 *Cre*^{ERT22};*Nr6a1*$^{flx/flx}$ embryos revealed a reduction in the number of rib-bearing thoracic elements compared to *Nr6a1*$^{flx/flx}$ when Tam was administered prior to E9.25. Scale bars are 1mm. Source data are provided as a Source Data file.
C. Quantification of the number of thoracic and lumbar elements in *Cre*^{ERT2};*Nr6a1*$^{flx/flx}$ and *Nr6a1*$^{flx/flx}$ embryos across all timepoints analysed. Shades of red represent *Cre*^{ERT2};*Nr6a1*$^{flx/flx}$ animals and shades of blue represent *Nr6a1*$^{flx/flx}$ animals. The number of biologically independent samples per genotype assessed: *Nr6a1*$^{flx/flx}$ Tam@6.5 n=22, Tam@7.5 n=32, Tam@8.5 n=38 and Tam@9.5 n=8; *Cre*^{ERT2};*Nr6a1*$^{flx/flx}$ Tam@6.5 n=16, Tam@7.5 n=28, Tam@8.5 n=32 and Tam@9.5 n=24. Error bars represent the mean with standard deviation. An unpaired two-tailed t-test was used for statistical comparisons between experimental and control embryos under the same treatment. Source data are provided as a Source Data file

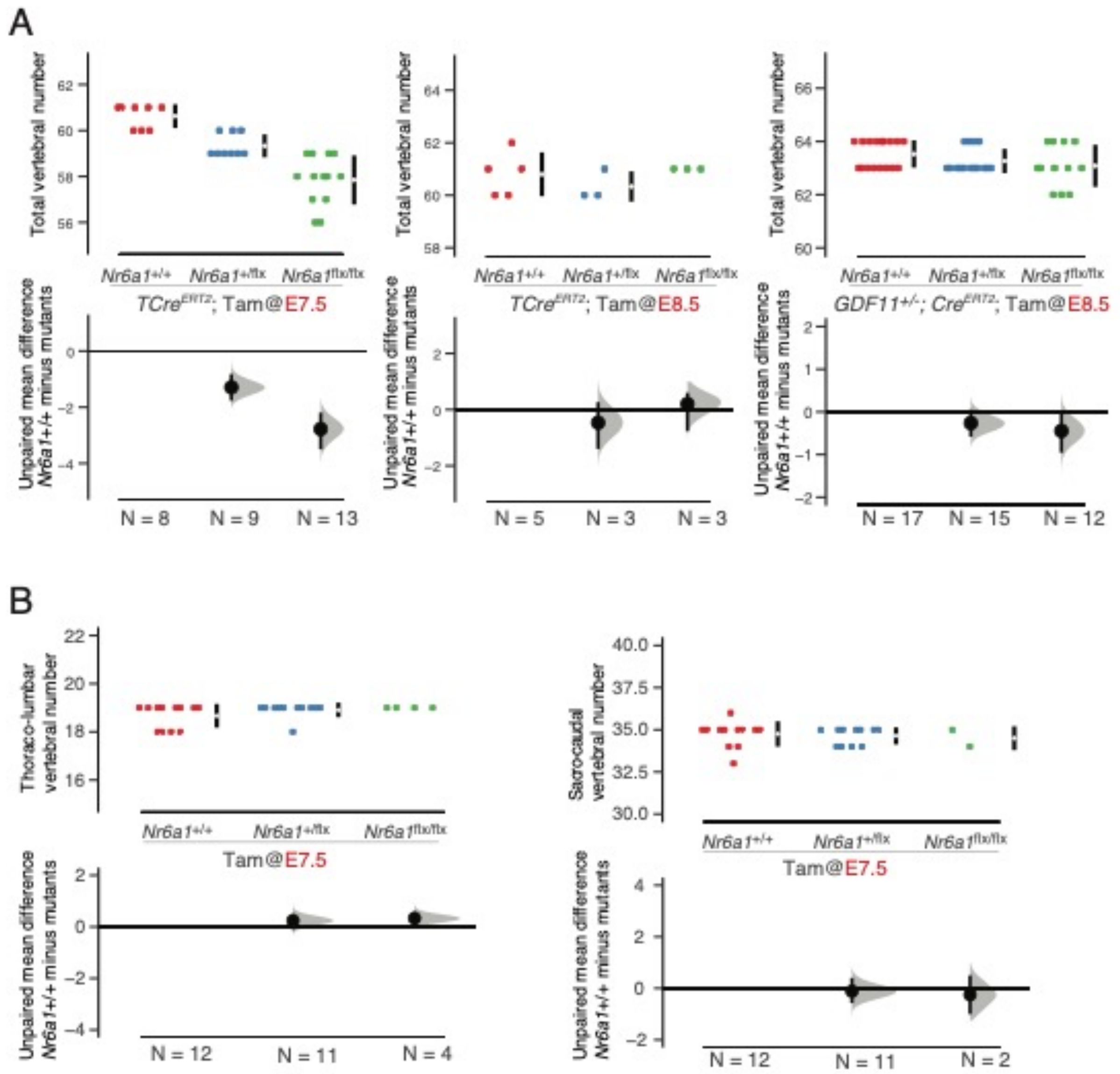

Supplementary Figure 3. Quantification of vertebral alterations following TCre^{ERT2}-mediated deletion of *Nr6a1*.

A. Quantification of total vertebral number following *TCre*^{ERT2}-mediated conditional deletion of *Nr6a1*. Tamoxifen (Tam) administration to pregnant dams at E7.5 resulted in a dose-dependent reduction in total vertebral number compared to wildtype, while Tam administration at E8.5 resulted in no difference between genotypes. Raw data is presented in the upper plots. Mean differences relative to *Nr6a1*$^{+/+}$ are presented in the lower plots as bootstrap sampling distributions. The mean difference for each genotype is depicted as a black dot and 95% confidence interval is indicated by the ends of the vertical error bar.

B. As a control, Tam administration at E7.5 in *TCre*^{ERT2}-negative animals showed no difference in thoraco-lumbar or sacro-caudal count. Raw data is presented in the upper plots. Mean differences relative to *Nr6a1*$^{+/+}$ are presented in the lower plots as bootstrap sampling distributions. The mean difference for each genotype is depicted as a black dot and 95% confidence interval is indicated by the ends of the vertical error bar.

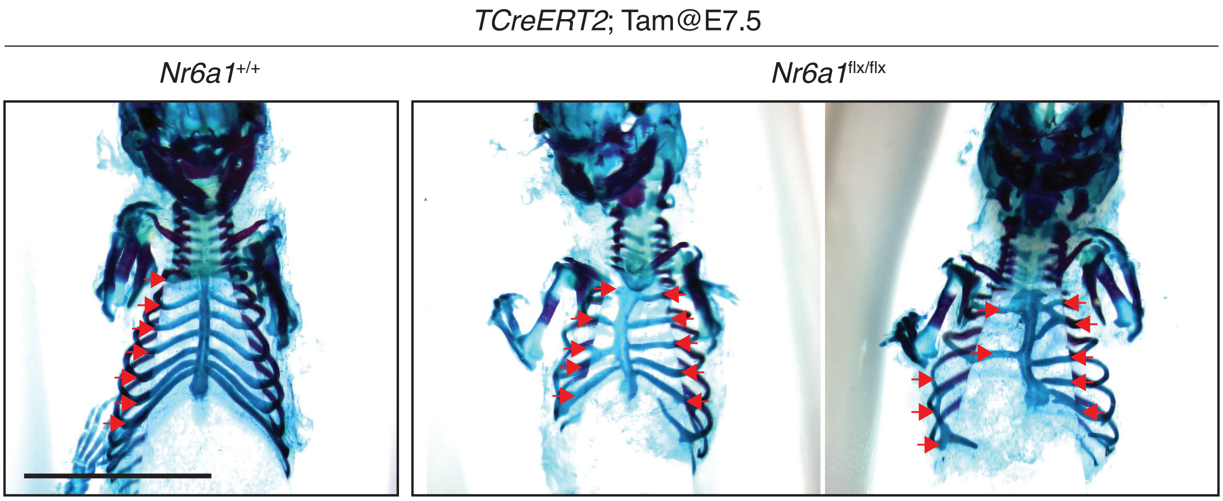

Supplementary Figure 4. Sternal rib reduction and malformation observed in *TCre*^{ERT2};*Nr6a1*$^{flx/flx}$ embryos.

Skeletal preparation of E16.5 embryos, ventral view, revealed a reduction in the number of sternal ribs (red arrows) and widespread rib fusions and malformations in *TCre*^{ERT2};*Nr6a1*$^{flx/flx}$ embryos (n=13/14 independent samples) compared to wildtype (no changes in n=8/8 independent samples). Tamoxifen (Tam) administration at E7.5.

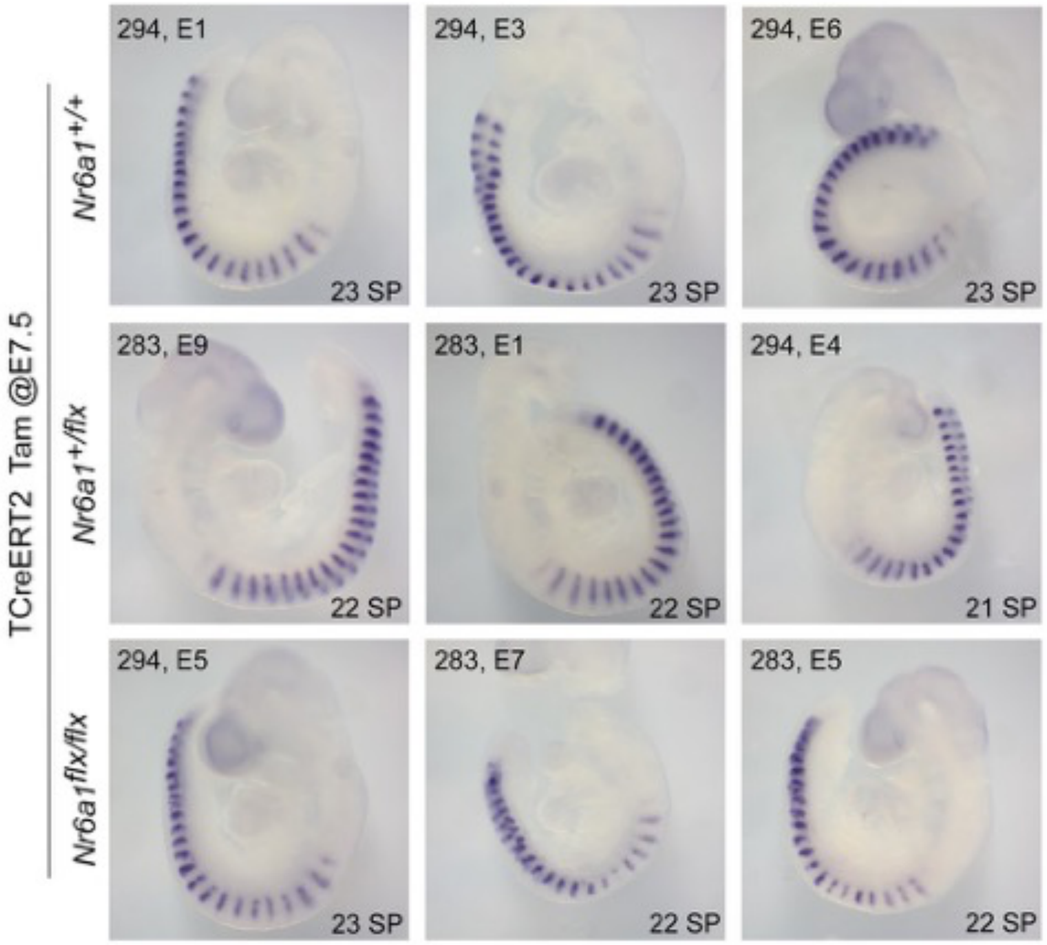

Supplementary Figure 5. Somite-staging of embryos used for quantitative PCR analysis

Following tailbud dissection for quantitative PCR analysis, the remainder of each embryo was processed for whole mount *in situ* hybridisation for the somite marker *Uncx4.1*. Each embryo shown is an independent biological sample. Somite pair (SP) number is indicated, comparisons are made between embryos +/- 1 somite. E=embryonic day; Tam=Tamoxifen.

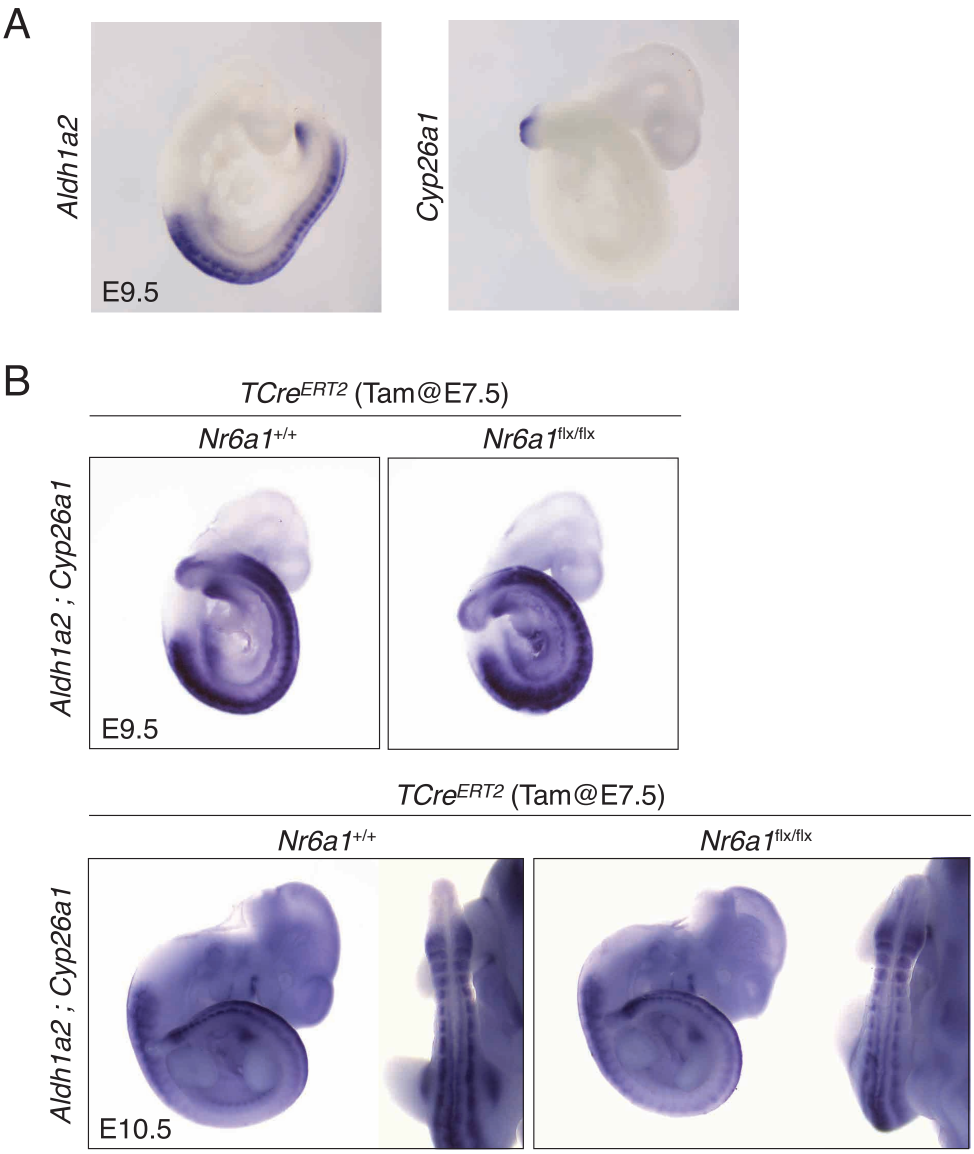

Supplementary Figure 6. Retinoic acid signalling analysis in *Nr6a1* conditional knockout embryos

A. Whole mount *in situ* hybridisation reveals non-overlapping expression of Retinoic acid synthesis enzyme *Aldh1a2* and RA degrading enzyme *Cyp26a1* in E9.5 WT embryos. Consistent expression patterns were observed in 3 biologically independent samples. E=embryonic day; Tam=Tamoxifen

B. (B-C) Dual *in situ* analysis of *Aldh1a2* and *Cyp26a1* in E9.5 (B) and E10.5 (D) WT and *TCreER*^{T2};*Nr6a1*$^{flx/flx}$ embryos showed no major spatio-temporal changes in either gene between genotypes. Consistent expression patterns were observed in 2 biologically independent samples/genotype/embryonic stage.

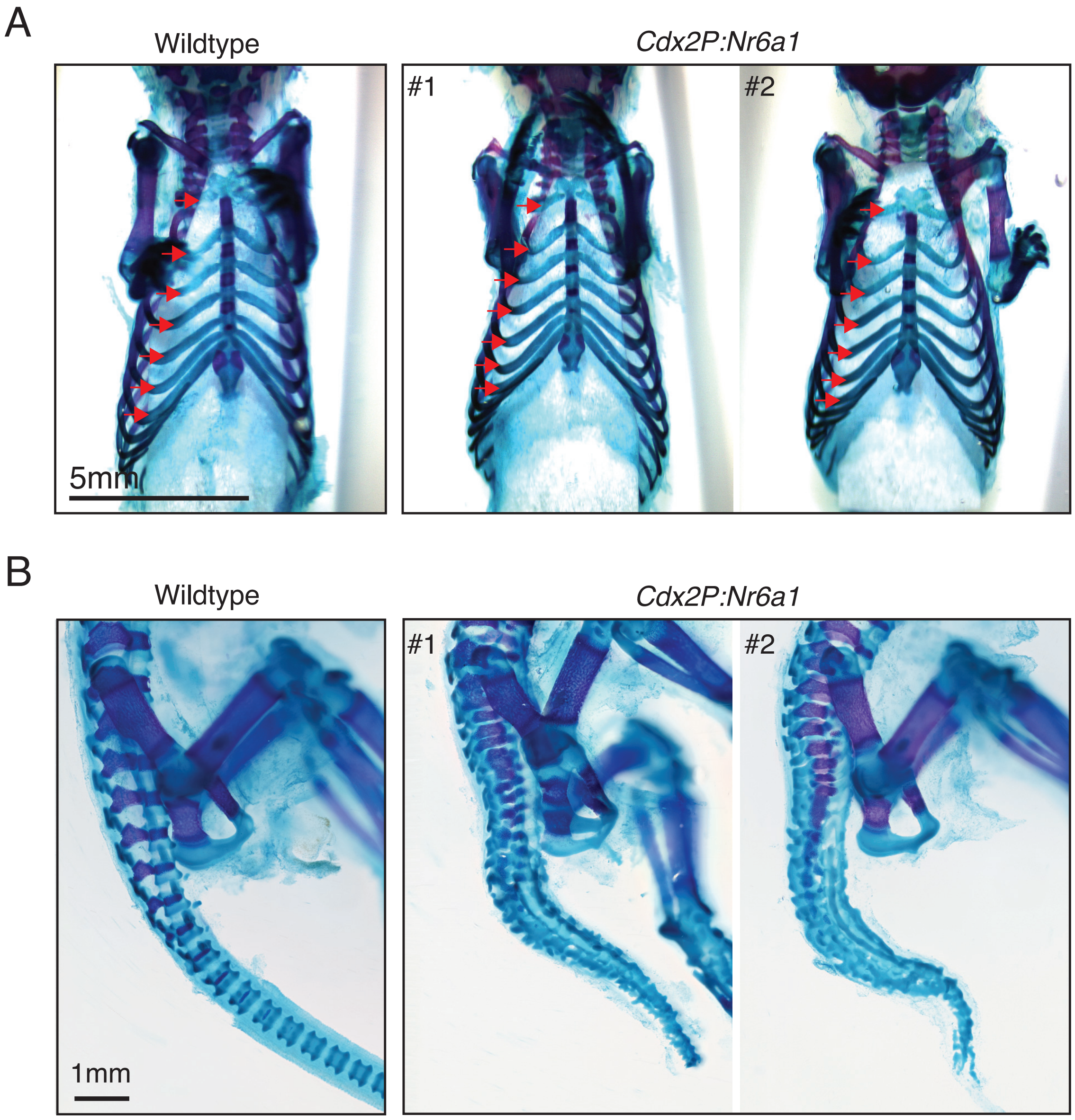

Supplementary Figure 7. Sternal rib and tail phenotype observed in *Cdx2P:Nr6a1* transgenic embryos.

A. Skeletal preparation of E18.5 embryos, lateral view, revealed a highly dysmorphic tail in *Cdx2P:Nr6a1* embryos, appearing to harbour many small and fused elements. Experimental numbers as per Supplementary Figure 7A.
B. Skeletal preparation of E18.5 embryos, ventral view, revealed no change in the number of sternal ribs (red arrow) in *Cdx2P:Nr6a1* embryos (n=2/2 independent samples) compared to wildtype embryos (n=8/8 independent samples).

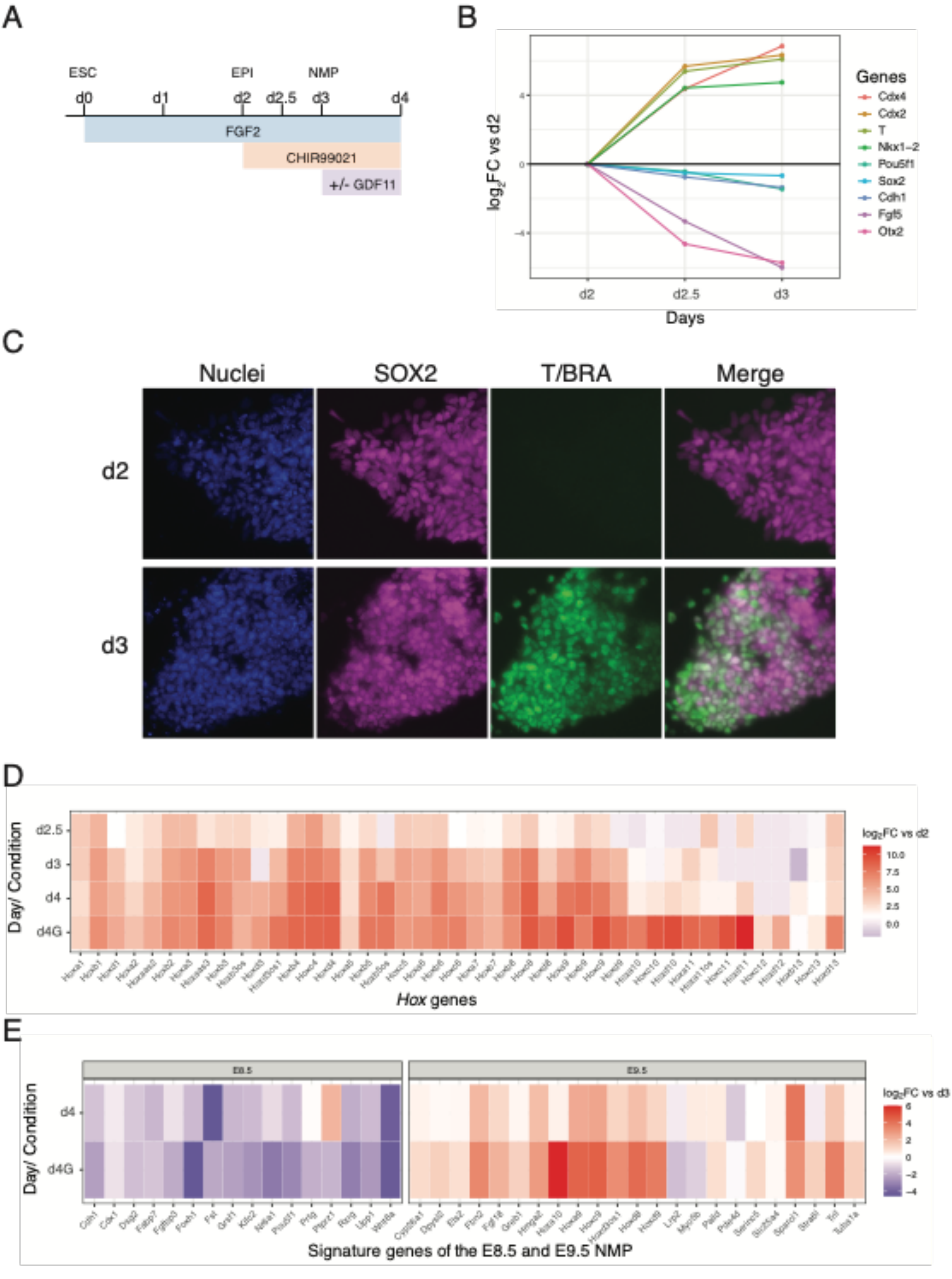

A
ESC
EPI
NMP
d0
d1
d2
d2.5
d3
d4
FGF2
CHIR99021
+/- GDF11
B
Genes
Cdx4
Cdx2
T
Nkx1−2
Pou5f1
Sox2
Cdh1
Fgf5
Otx2
log2FC vs d2
Days
C
Nuclei
SOX2
T/BRA
Merge
d2
d3
D
Day/ Condition
d2.5
d3
d4
d4G
log2FC vs d2
Hox genes
E
E8.5
E9.5
Day/ Condition
d4
d4G
log2FC vs d3
Signature genes of the E8.5 and E9.5 NMP

Supplementary Figure 8. Characterisation of wildtype *in vitro* differentiation kinetics

A. Schematic representation of the embryonic stem cell (ESC) *in vitro* differentiation protocol for generating epiblast-like (EPI) and neuromesodermal-like (NMP) cells over time. d = day of differentiation.
B. RNAseq analysis of d2, d2.5 and d3 transcriptomes was used to monitor differentiation progression. Expression of the epiblast-related genes such as *Pou5f1*, *Sox2*, *Cdh1*, *Fgf5* and *Otx2* declined once d2 EPI cells were treated with CHIR99021, whereas expression of the NMP-enriched genes, such as *Cdx4*, *Cdx2*, *T/Bra* and *Nkx1-2* increased. n= 3 technical replicates per day. Source data are provided as a Source Data file.
C. In-well immunofluorescence of the canonical NMP markers, Sox2 (magenta) and T/Bra (green) was performed on d2 and d3 of differentiation, nuclei (blue) stained with DAPI, n=3 independent replicate experiments. T/Bra was not detected in d2 cells. Sox2 and T/Bra double positive cells (white) were observed throughout colonies on d3.
D. Complete collinear activation of *Hox* genes requires the sequential addition of CHIR99021 and Gdf11. Results are presented as a log2-transformed fold change of expression in d2.5, d3, d4 and d4G cells relative to d2. n= 3 technical replicates per day and per condition. Source data are provided as a Source Data file.
E. Temporal change in expression of the temporal signature genes for E8.5 and E9.5 NMPs[20]. Results are presented as a log2-transformed fold change in d4 and d4G cells relative to d3 NMPs, n= 3 sample per cell type.

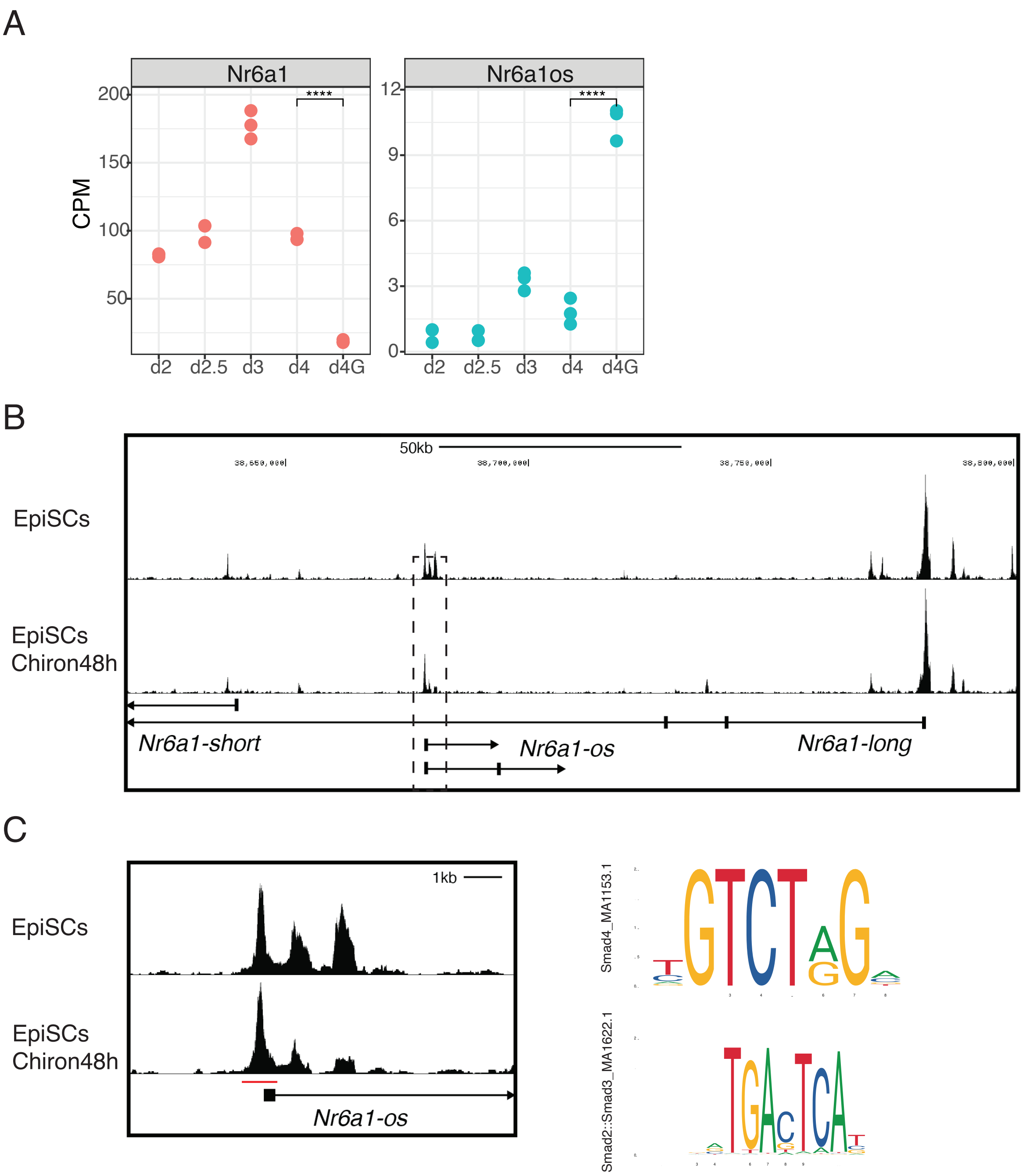

Supplementary Figure 9. *In vitro* regulation of *Nr6a1* and *Nr6a1os*

A. *Nr6a1* and *Nr6a1os* expression analysis over the course of *in vitro* differentiation as schematised. RNA-seq data is presented as counts per million (CPM) and statistical changes in gene expression downstream of Gdf11 addition was performed using edgeR. ****FDR< 0.0001.

B. Identification of genomically-accessible regions surrounding the *Nr6a1* locus in epiblast stem cells (EpiSCs) with or without Chiron treatment for 48h. ATAC-seq data derived from[57].

C. The promoter region of *Nr6a1os*, framed in Supplementary Figure 7A. The red line indicates the genomic region used for transcription factor binding profiles using the JASPAR database (https://jaspar.genereg.net), revealing multiple predicted Smad binding sites.

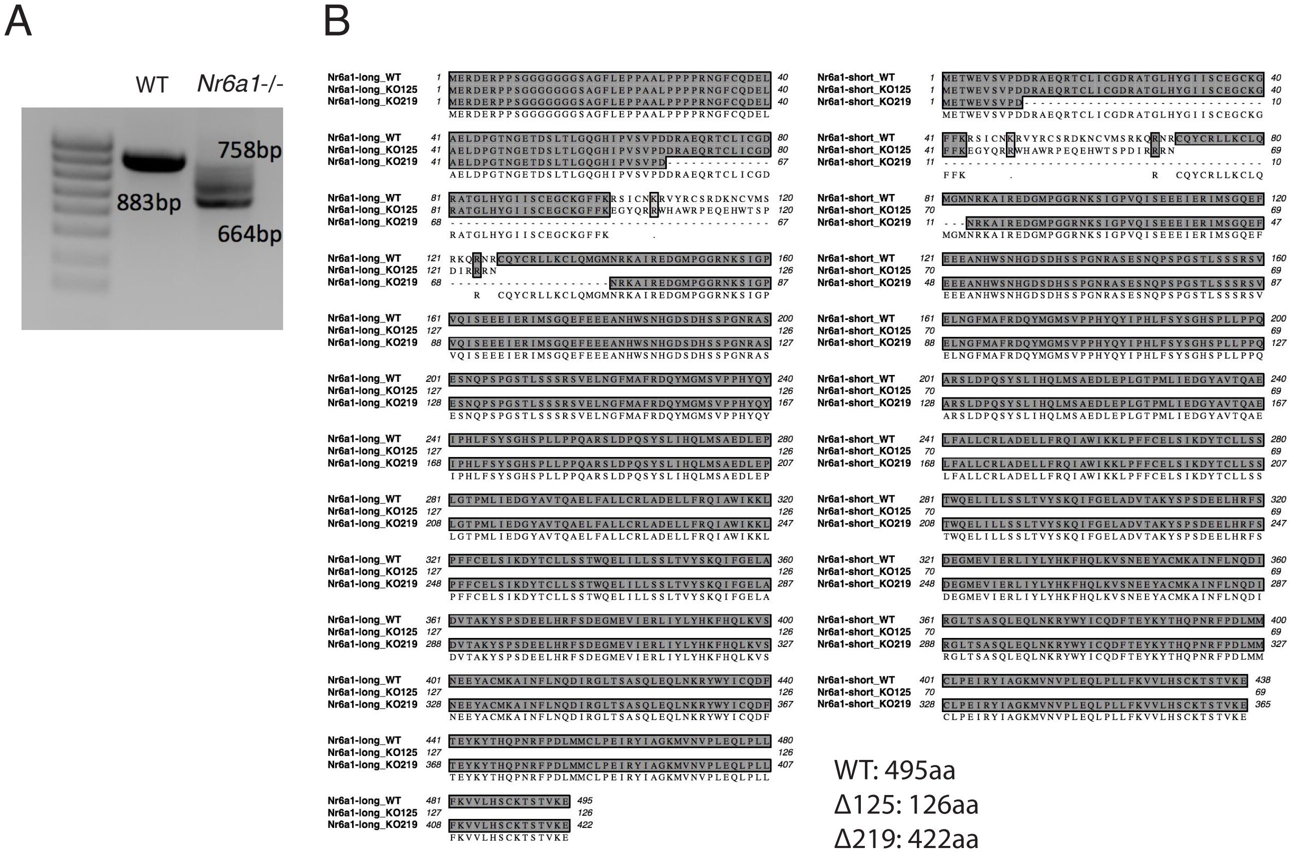

Supplementary Figure 10. Genomic deletion of *Nr6a1*$^{-/-}$ in mouse embryonic stem cells

A. PCR amplification using primers external to the PAM site reveal successful genomic deletion in the clonal Nr6a1$^{-/-}$ ESC line used for analysis, compared to the expected wildtype product of 883bp. n= 2 independent PCR replicates.

B. The alignments of wildtype *Nr6a1* with Δ219 and Δ125 *Nr6a1*. The Δ125 mutant allele causes a premature truncation within the DNA binding domain, removing 369 amino acids relative to the wildtype protein. The Δ219 allele causes a 73 amino acid deletion, removing almost the entirety of the DNA binding domain. The similar molecular outcomes observed for loss-of-function *in vitro* and *in vivo* datasets supports this being a loss-of-function line, though this remains to be formally proven.

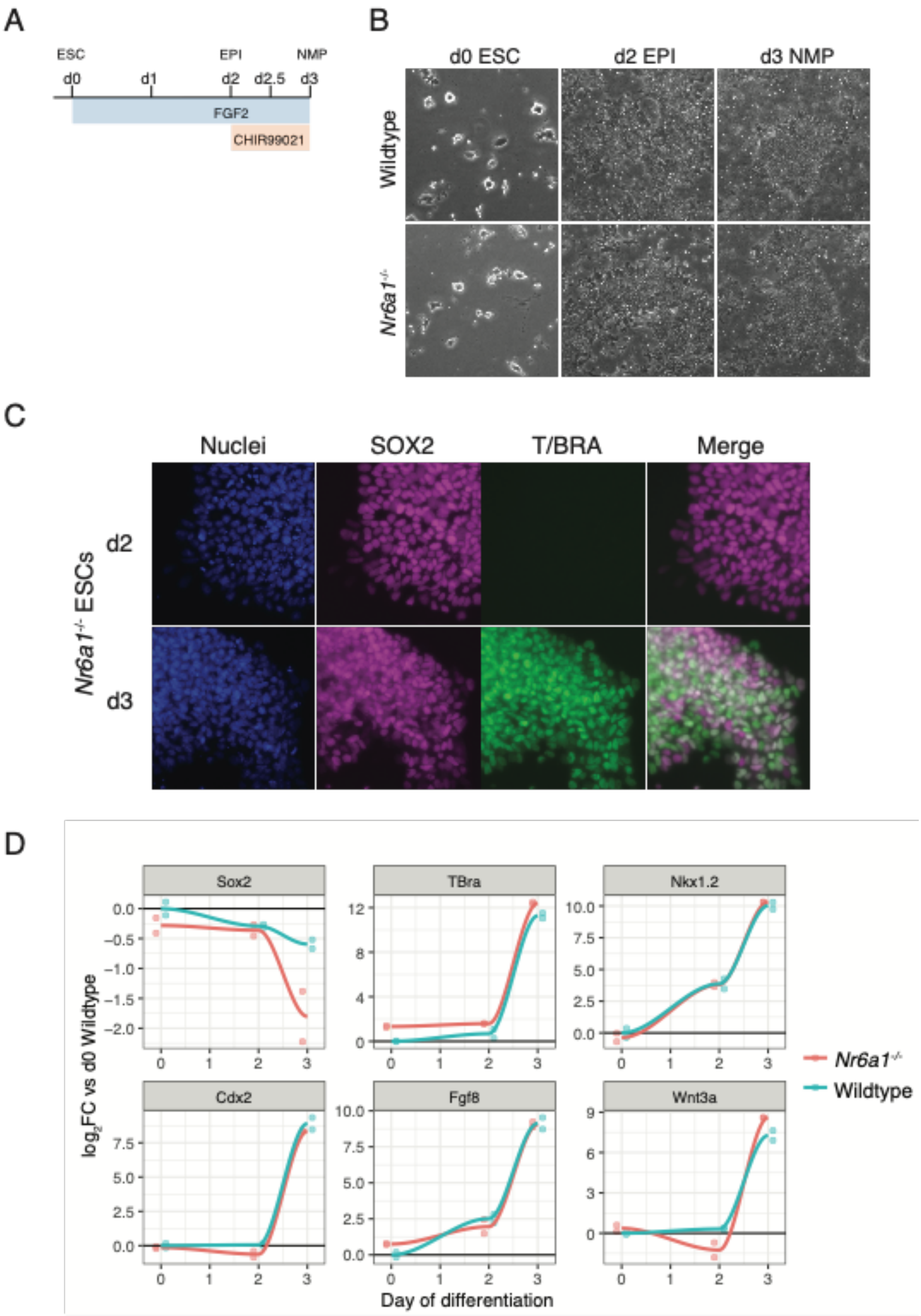
A
ESC
EPI
NMP
d0
d1
d2
d2.5
d3
FGF2
CHIR99021
B
d0 ESC
d2 EPI
d3 NMP
Wildtype
Nr6a1-/-
C
Nuclei
SOX2
T/BRA
Merge
Nr6a1-/- ESCs
d2
d3
D
Sox2
TBra
Nkx1.2
Cdx2
Fgf8
Wnt3a
log2FC vs d0 Wildtype
Day of differentiation
Nr6a1-/-
Wildtype

Supplementary Figure 11. Successful *in vitro* generation of NMPs from *Nr6a1*$^{-/-}$ ESCs

A. Schematic of *in vitro* differentiation conditions that apply to panels B and C. Embryonic Stem Cell (ESC); Epiblast-like cell (EPI); neuromesodermal-like cell (NMP).
B. At a morphological level, *Nr6a1*$^{-/-}$ cells were indistinguishable from wildtype over the course of *in vitro* differentiation, n=>3 independent experimental replicates.
C. In-well immunofluorescence of the canonical NMP markers, Sox2 (magenta) and T/Bra (green) was performed on d2 and d3 of differentiation, nuclei (blue) stained with DAPI, n=3 independent replicate experiments. T/Bra was not detected in d2 cells. Sox2 and T/Bra double positive cells (white) were observed throughout colonies on d3.
D. At a molecular level, *Nr6a1*$^{-/-}$ cells were able to generate NMP-like cells with near-identical kinetics to wildtype, as assessed by qPCR analysis of known NMP marker genes. The results of quantitative PCR analysis is presented as a $\log_2$ fold change (FC) relative to expression of each gene in d0 WT cells. Source data are provided as a Source Data file.

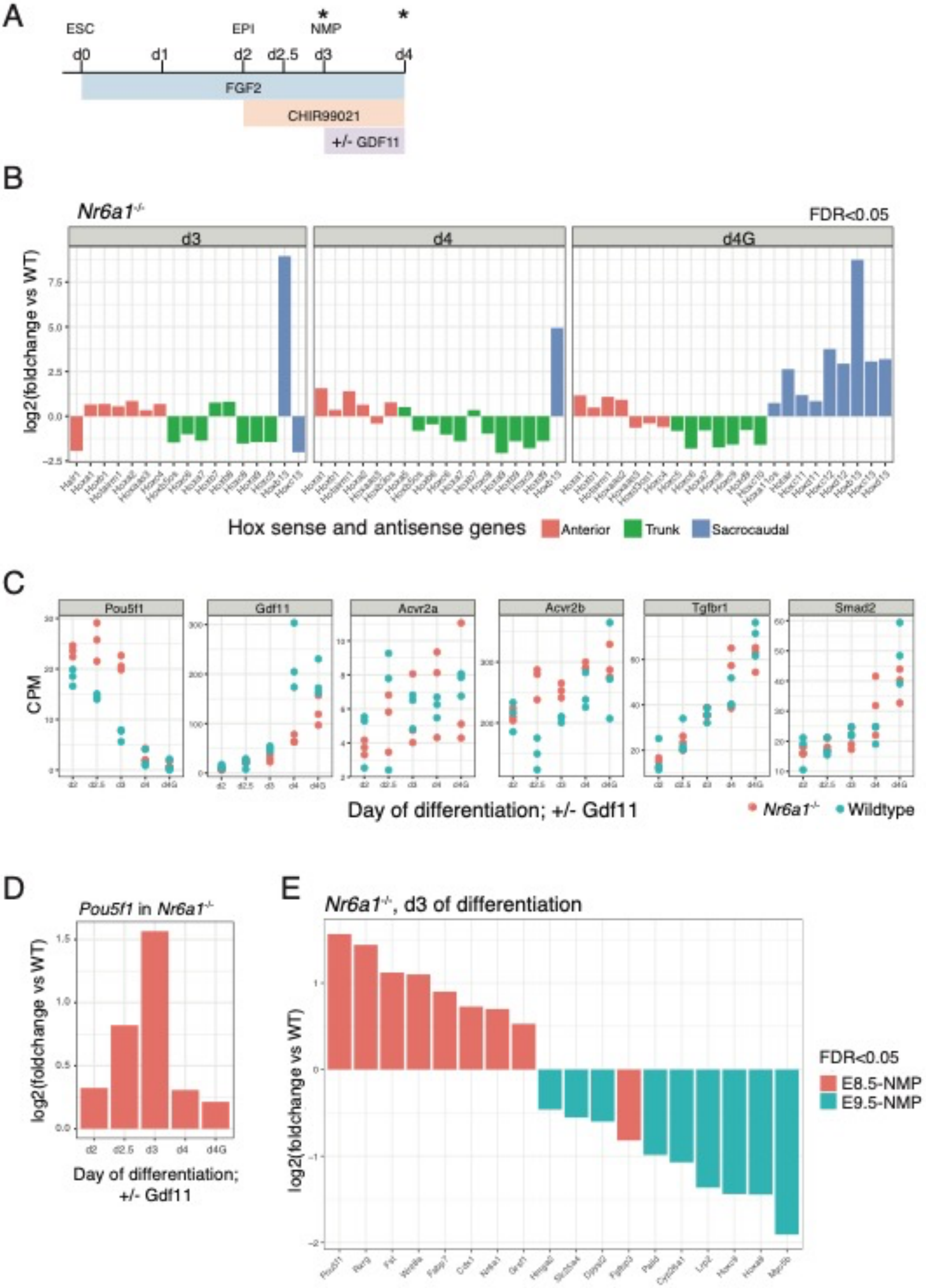
A
ESC
EPI
NMP
d0
d1
d2
d2.5
d3
d4
FGF2
CHIR99021
+/- GDF11
B
Nr6a1-/-
FDR<0.05
d3
d4
d4G
log2(foldchange vs WT)
Hox sense and antisense genes
Anterior
Trunk
Sacrocaudal
C
Pou5f1
Gdf11
Acvr2a
Acvr2b
Tgfbr1
Smad2
CPM
Day of differentiation; +/- Gdf11
Nr6a1-/-
Wildtype
D
Pou5f1 in Nr6a1-/-
log2(foldchange vs WT)
Day of differentiation;
+/- Gdf11
E
Nr6a1-/-, d3 of differentiation
log2(foldchange vs WT)
FDR<0.05
E8.5-NMP
E9.5-NMP

Supplementary Figure 12. Opposing regulation of trunk vs. caudal *Hox* genes by Nr6a1

A. Schematic of *in vitro* differentiation conditions that apply to panels B-D. Embryonic Stem Cell (ESC); Epiblast-like cell (EPI); neuromesodermal-like cell (NMP).
B. RNAseq analysis of *Oct4*/*Pou5f1* across differentiation, presented as a log2-transformed fold change in *Nr6a1*$^{-/-}$ samples relative to wildtype. A significant upregulation of *Oct4* in *Nr6a1*$^{-/-}$ cells relative to wildtype was observed at Day(d)2.5 and d3 (FDR < 0.05). Source data are provided as a Source Data file.
C. RNAseq analysis of E8.5-NMP and E9.5-NMP signature genes (gene lists from Gouti et al., 2017) at d3 of differentiation. Results are presented as a log2-transformed fold change in *Nr6a1*$^{-/-}$ cells relative to wildtype, n=3/genotype, only genes with FDR<0.05 displayed. E8.5-NMP gene expression levels are quantitatively enhanced and E9.5-NMP gene expression levels reduced in *Nr6a1*$^{-/-}$ cells at d3 relative to WT.
D. RNAseq analysis of *Nr6a1*$^{-/-}$ in vitro NMPs revealed opposing regulation of trunk vs posterior *Hox* genes by Nr6a1. Results are presented as a log2-transformed fold change in *Nr6a1*$^{-/-}$ cells relative to wildtype, n=3/genotype. Only those *Hox* genes with a false discovery rate (FDR) < 0.05 are displayed, and are colour-coded based on the axial region where the Hox protein functions. Source data are provided as a Source Data file.
E. RNAseq analysis of *Oct4*/*Pou5f1* and various components of the Gdf11 signalling pathway in *Nr6a1*$^{-/-}$ (red) and wild type (blue) cells. Data represented as counts per million (CPM). Source data are provided as a Source Data file.

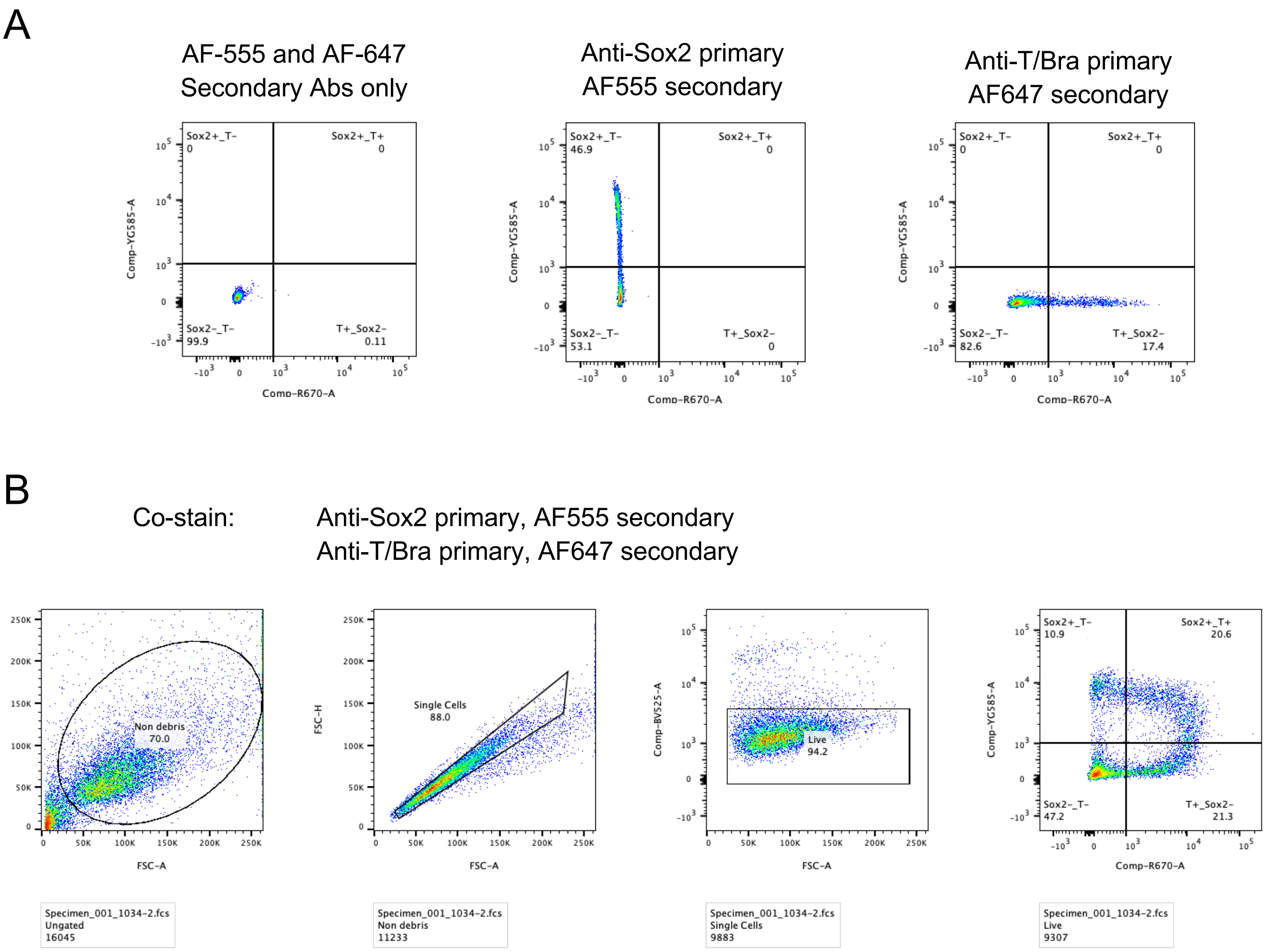

Supplementary Figure 13. Flow cytometry gating parameters

A. Control staining's used to establish Flow cytometry gating parameters.

B. Representative plots depicting final gating parameters for experimental samples. Cell suspensions for analysis were gated to remove debris, isolate single cells, remove non-viable cells before detection of Sox2 and T/Bra secondary fluorophores.

Supplementary Table 1

Resources table

REAGENT or RESOURCE	SOURCE	IDENTIFIER
**Antibodies**		
Anti-Digoxigenin Fab fragments Antibody, AP Conjugated	Roche	Cat# 11093274910, RRID:AB_514497
Rat monoclonal anti-Sox2 (Btjce)	eBiosciences	14-9811-82
Rabbit monoclonal anti-T/Brachyury	Abcam	ab209665
Goat anti-rat IgG, AF555	Invitrogen	A48263
Goat anti-rabbit IgG	Invitrogen	A32733
**Chemicals, peptides, and recombinant proteins**		
Human FGF-2 IS	Miltenyi Biotec	Cat# 130-1-4925
StemMACS^{TM} CHIR99021	Miltenyi Biotec	Cat# 130-103-926
Human GDF-11	Miltenyi Biotec	Cat# 130-105-776
**Experimental models: Cell lines**		
Wildtype mouse ES cell line - Bruce4	(Abbondanzo et al., 1993)	N/A
Nr6a1$^{-/-}$ mESCs	This study	N/A
**Experimental models: Organisms/strains**		
Mouse *Nr6a1*$^{flx/flx}$	(Lan et al., 2003)	N/A
Mouse *TCreER*^{T2}	(Anderson et al., 2013)	N/A
Mouse *Gdf11*$^{+/-}$	(McPherron et al., 1999)	N/A
Mouse C57BL/6J	Monash Animal Research Platform	N/A

Oligonucleotides		
Nr6a1-long-clone-Fwd: TGGAAGACCAGGACGACGACTA	This study	N/A
Nr6a1-short-clone-Fwd: GTCATCAAGCGGAGTTTACCCTG	This study	N/A
Nr6a1-clone-Rev: CTGCTCCACAGTCTCCATCTTG	This study	N/A
Nr6a1-qPCR-Fwd: CACCAGGCTCCACACTATCA	This study	N/A
Nr6a1-qPCR-Rev: GATCCCTGAATGCCATGAAT	This study	N/A
Sox2-qPCR-Fwd: GCACATGAACGGCTGGAGCAACG	This study	N/A
Sox2-qPCR-Rev: TGCTGCGAGTAGGACATGCTGTAG	This study	N/A
T/Bra-qPCR-Fwd: GGCTGTTGGGTAGGGAGTCA	This study	N/A
T/Bra-qPCR-Rev: GGAACATCCTCCTGCCGTTCTT	This study	N/A
Nkx1.2-qPCR-Fwd: CAGCTGGTGCCAGATGAGATTAGA	This study	N/A
Nkx1.2-qPCR-Rev: CCTGAGAGGGTACCTTTCCCACTT	This study	N/A
Cdx2-qPCR-Fwd: GACTTCCTGTCCCTTCCCTCGTCT	(Lau and Marikawa, 2014)	N/A
Cdx2-qPCR-Rev: CCTCCCGACTTCCCTTCACCATAC	(Lau and Marikawa, 2014)	N/A
Fgf8-qPCR-Fwd: TCTCCAGCACGATCTCTGTGAA	(Mariani et al., 2008)	N/A
Fgf8-qPCR-Rev: GGAAGCTAATTGCCAAGAGCAA	(Mariani et al., 2008)	N/A
Wnt3a-qPCR-Fwd: GCCACAAGAGCTTCCTGATTGGTA	(Lau and Marikawa, 2014)	N/A
Wnt3a-qPCR-Rev:	(Lau and Marikawa, 2014)	N/A

CCAGGCAGAAGACAGTCAGTCACC		
Oct4-qPCR-Fwd: TAGGTGAGCGCTCTTTCCAC	This study	N/A
Oct4-qPCR-Rev: GCTTAGCCAGGTTCGAGGAT	This study	N/A
Hoxa10-qPCR-Fwd: GGAAGCATGGACATTCAGGT	(Kondrashov et al., 2011)	N/A
Hoxa10-qPCR-Rev: CCAGGCAAGCAAGACCTTAG	(Kondrashov et al., 2011)	N/A
Hoxc10-qPCR-Fwd: GAGCGGAAGGAAGAAGAGGT	(Kondrashov et al., 2011)	N/A
Hoxc10-qPCR-Rev: GATCCGATTCTCTCGGTTCA	(Kondrashov et al., 2011)	N/A
Hoxc11-qPCR-Fwd: GCTTCTTCGACAACGCCTAC	(Kondrashov et al., 2011)	N/A
Hoxc11-qPCR-Rev: GAGCTGGGATTCGTGTTCTC	(Kondrashov et al., 2011)	N/A
Hoxd11-qPCR-Fwd: TGAACGACTTTGACGAGTGC	(Kondrashov et al., 2011)	N/A
Hoxd11-qPCR-Rev: GGTTGGAGGAGTAGGGGAAA	(Kondrashov et al., 2011)	N/A
Hoxc12-qPCR-Fwd: GGCTACCCACAGCCCTATCT	(Kondrashov et al., 2011)	N/A
Hoxc12-qPCR-Rev: TCGCCGTGTAGTCGTACTTG	(Kondrashov et al., 2011)	N/A
Hoxd12-qPCR-Fwd: GCATGAAACAGGAGCCTAGC	(Kondrashov et al., 2011)	N/A
Hoxd12-qPCR-Rev: CCTTTCCTTCCTGCAGAGTG	(Kondrashov et al., 2011)	N/A
Hoxa13-qPCR-Fwd: CCTCTGGAAGTCCACTCTGC	(Kondrashov et al., 2011)	N/A
Hoxa13-qPCR-Rev: CCTCCGTTTGTCCTTGGTAA	(Kondrashov et al., 2011)	N/A
Hoxb13-qPCR-Fwd: AGGTGAACAGAACCCACCAG	(Kondrashov et al., 2011)	N/A

Hoxb13-qPCR-Rev: TTGCGCCTCTTGTCCTTAGT	(Kondrashov et al., 2011)	N/A
Hoxc13-qPCR-Fwd: CCTCTACAGCCCGAAGTGAG	(Kondrashov et al., 2011)	N/A
Hoxc13-qPCR-Rev: CTGGAACCAGATGGTGACCT	(Kondrashov et al., 2011)	N/A
Hoxd13-qPCR-Fwd: CGACATGGTGTCCACTTTTG	(Kondrashov et al., 2011)	N/A
Hoxd13-qPCR-Rev: TGGTGTAAGGCACCCTTTTC	(Kondrashov et al., 2011)	N/A
Pol2a-qPCR-Fwd: GCACCACGTCCAATGATAT	This study	N/A
Pol2a-qPCR-Rev: GTGCGGCTGCTTCCATAA	This study	N/A
sgNr6a1-1: TGGAAAGGACGAAACACC**GTTGTTCAGCTCGATCATC**GTTTAAGAGCTATGCTGGAAACA	This study	N/A
sgNr6a1-2: TGGAAAGGACGAAACACC**GTCTCCAGATGGGCATGAAC**GTTTAAGAGCTATGCTGGAAAC	This study	N/A
sgRNA_HDRstep1-Fwd: TGTTTTAAAATGGACTATCATATGCTTACCGTAACTTGAAAGTATTTCGATTTCTTGGCTTTATATATCTTGTGGAAAGGACGAAACACC	(Arbab and Sherwood, 2016)	N/A
sgRNA_HDRstep1-Rev: GTTGATAACGGACTAGCCTTATTTAAACTTGCTATGCTGTTTCCAGCATAGCTCTTAAAC	(Arbab and Sherwood, 2016)	N/A
sgRNA_HDRstep2-Fwd: GTACAAAATACGTGACGTAGAAAGTAATAATTTCTTGGGTAGTTTGCAGTTTTAAAATTATGTTTTAAAATGGACTATCATATGCTTACC	(Arbab and Sherwood, 2016)	N/A
sgRNA_HDRstep2-Rev:	(Arbab and Sherwood, 2016)	N/A

ATTTTAACTTGCTATTTCTAGCTCTAA AACAAAAAAGCACCGACTCGGTGCCA CTTTTTCAAGTTGATAACGGACTAGCC TTATTTAAAC		
sgRNA_HDRstep3-Fwd: CGATACAAGGCTGTTAGAGAGATAAT TAGAATTAATTTGACTGTAAACACAA AGATATTAGTACAAAATACGTGACGT AGAAAGTAATAA	(Arbab and Sherwood, 2016)	N/A
sgRNA_HDRstep3-Rev: TCAATGTATCTTATCATGTCTGCTCGA TTTTAACTTGCTATTTCTAGCTCTAAA ACAAAA	(Arbab and Sherwood, 2016)	N/A
LGT-Nr6a1e4-Fwd: CTCAGGGGCATGTTTTGTT	This study	N/A
LGT-Nr6a1e4-Rev: TCTCTGTGTAGCTCTGACTGA	This study	N/A
Gdf11-GT-WT-Fwd: GCTACAAGGCCAACTACTGCTC	This study	N/A
Gdf11-GT-WT-Rev: AATCTGCTGCTTGTCATTGAAG	This study	N/A
Gdf11-GT-Mut-Fwd: AGGATCTCCTGTCATCTCACCTTGCTC CTG	This study	N/A
Gdf11-GT-Mut-Rev: AAGAACTCGTCAAGAAGGCGATAGAA GGCG	This study	N/A
Nr6a1-GT-Flx-Fwd: CGAACTCAGAAATCCACC	(Lan et al., 2003)	N/A
Nr6a1-GT-Flx-Rev: AATCACAAACACCACAACTC	(Lan et al., 2003)	N/A
Nr6a1-GT-Lox-Fwd: CCAATTCCCCCAAAGTGTC	(Lan et al., 2002)	N/A
Nr6a1-GT-Lox-Rev: CAGGTCGAGGGACCTATAAC	(Lan et al., 2002)	N/A
TCreERT2-GT-Fwd: GGGACCCATTTTTCTCTTCC	(Anderson et al., 2013)	N/A

TCreERT2-GT-Rev: CCATGAGTGAACGAACCTGG	(Anderson et al., 2013)	N/A
Recombinant DNA		
Cdx2P:Nr6a1 transgenic construct	This paper	N/A
sgPal7(p2Tol-U6-sgPal7-HygR)	(Arbab et al., 2015)	RRID:Addgene71484
spCas9-BlastR(pCBhCas9-BlastR)	(Arbab et al., 2015)	RRID:Addgene71489
Software and algorithms		
STAR	(Dobin et al., 2013)	RRID:SCR_004463
Degust	(Powell, 2015)	https://degust.erc.monash.edu/
R studio cloud	RStudio	https://rstudio.cloud/
dabestr	(Ho et al., 2019)	https://github.com/ACCLAB/dabestr
