# Supplementary material for: Nr6a1 controls Hox expression dynamics and is a master regulator of vertebrate trunk development
Source: Nat Commun. 2022 Dec 15;13:7766. doi: 10.1038/s41467-022-35303-4 (PMC9755267; doi:10.1038/s41467-022-35303-4)
Supplement: Supplementary file 3 — Description of Additional Supplementary Files [file 41467_2022_35303_MOESM3_ESM.pdf]

Description of Additional Supplementary Files

File name: **Supplementary Data 1**
Description: ToppGene and DAVID analysis of *Nr6a1*$^{-/-}$ embryos compared to controls.

File name: **Supplementary Data 2**
Description: Differentially expressed genes (FDR<0.05) uniquely identified in *Cdx2P:Nr6a1* tailbuds, uniquely identified in *Gdf11*$^{-/-}$ tailbuds, or common to both *Cdx2P:Nr6a1* and *Gdf11*$^{-/-}$ tailbuds.
