# Supplementary material for: Nr6a1 controls Hox expression dynamics and is a master regulator of vertebrate trunk development
Source: Nat Commun. 2022 Dec 15;13:7766. doi: 10.1038/s41467-022-35303-4 (PMC9755267; doi:10.1038/s41467-022-35303-4)
Supplement: Supplementary file 6 — Reporting Summary [file 41467_2022_35303_MOESM6_ESM.pdf]

nature portfolio

Corresponding author(s): Edwina McGlinn

Last updated by author(s): 17 November, 2022

# Reporting Summary

Nature Portfolio wishes to improve the reproducibility of the work that we publish. This form provides structure for consistency and transparency in reporting. For further information on Nature Portfolio policies, see our Editorial Policies and the Editorial Policy Checklist.

## Statistics

For all statistical analyses, confirm that the following items are present in the figure legend, table legend, main text, or Methods section.

| n/a | Confirmed | |
|---|---|---|
| ☐ | ☒ | The exact sample size ($n$) for each experimental group/condition, given as a discrete number and unit of measurement |
| ☐ | ☒ | A statement on whether measurements were taken from distinct samples or whether the same sample was measured repeatedly |
| ☐ | ☒ | The statistical test(s) used AND whether they are one- or two-sided<br>*Only common tests should be described solely by name; describe more complex techniques in the Methods section.* |
| ☒ | ☐ | A description of all covariates tested |
| ☒ | ☐ | A description of any assumptions or corrections, such as tests of normality and adjustment for multiple comparisons |
| ☐ | ☒ | A full description of the statistical parameters including central tendency (e.g. means) or other basic estimates (e.g. regression coefficient) AND variation (e.g. standard deviation) or associated estimates of uncertainty (e.g. confidence intervals) |
| ☐ | ☒ | For null hypothesis testing, the test statistic (e.g. $F$, $t$, $r$) with confidence intervals, effect sizes, degrees of freedom and $P$ value noted<br>*Give P values as exact values whenever suitable.* |
| ☒ | ☐ | For Bayesian analysis, information on the choice of priors and Markov chain Monte Carlo settings |
| ☒ | ☐ | For hierarchical and complex designs, identification of the appropriate level for tests and full reporting of outcomes |
| ☐ | ☒ | Estimates of effect sizes (e.g. Cohen's $d$, Pearson's $r$), indicating how they were calculated |

*Our web collection on statistics for biologists contains articles on many of the points above.*

## Software and code

Policy information about availability of computer code

| | |
|---|---|
| Data collection | Software:<br>RStudio (R version 4.0.1)<br>Degust (https://degust.erc.monash.edu/)<br>Microarray analysis was performed using Affymetrix GeneChip GeneChip Mouse Genome 430 2.0 Arrays processed with the GeneChip Fluidics Station 450 and scanned with a GeneChip Scanner 3000 7G |
| Data analysis | Quantitative PCR was performed using the 96*96 BioMark Fluidigm format. Raw Ct values were analysed using a modified version of the qPCR-Biomark script (https://github.com/jpouch/qPCR-Biomark). Only Ct-values in the optimal range for the Biomark system of 6-25 were used for further analysis. All genes were first normalised against the mean raw Ct-values of five housekeeping gene probes yielding ΔCt values, then normalised against the wildtype condition yielding ΔΔCt values<br><br>For RNA-seq analysis, bulk RNA-seq reads are aligned to the mm10 reference (GRCm38, Ensembl v93) genome using STAR (v020201). UMI duplicates are identified using the markdupes function from the je package (v1.2). We count transcriptomic reads using featureCounts (v1.5.2). Differential gene expression between control and mutants were performed using edgeR in the open platform Degust (https://degust.erc.monash.edu/). Here we used false discovery rate (FDR), the adjusted p value, to display significance of the differential gene expression between the controls and the mutants. Genes with a FDR < 0.05 were considered to be significantly differential expressed in the mutants.<br><br>Microarray CEL files were analyzed using RMA (Irizarry et al., 2003) and limma (Smyth, 2004) in the R statistical environment. Following alignment and annotation of genes, the Microarray data was filtered to export differentially expressed genes with an adjusted p-value or false discovery rate of 0.05 or lower and a fold change less than or equal to -2 and greater than or equal to 2 (log2FC less than or equal to -1 and greater than or equal to 1). |

Vertebral formulae - CMVCreERT2 analysis: For statistical analysis and data visualisation of vertebral numbers, Graph Pad Prism 9.3.1 (471) was used. A two-tailed T-test was employed. Error bars represent the mean with standard deviation.

Vertebral formulae - TCreERT2 analysis: For statistical analysis and data visualisation of vertebral numbers, R-package “Data Analysis using Bootstrap-Coupled ESTimation” (dabestr) was used. To determine mean differences to the respective shared control, 5000 bootstrap samples were taken and the confidence interval was bias-corrected and accelerated. In the visualisation, 95% confidence interval is indicated by the ends of the vertical error bar and the sampling error distribution is diagrammed as a grey filled curve. The codes are available at https://github.com/ACCLAB/dabestr

For manuscripts utilizing custom algorithms or software that are central to the research but not yet described in published literature, software must be made available to editors and reviewers. We strongly encourage code deposition in a community repository (e.g. GitHub). See the Nature Portfolio guidelines for submitting code & software for further information.

## Data

Policy information about availability of data

All manuscripts must include a data availability statement. This statement should provide the following information, where applicable:

- Accession codes, unique identifiers, or web links for publicly available datasets
- A description of any restrictions on data availability
- For clinical datasets or third party data, please ensure that the statement adheres to our policy

The raw data associated with the microarray experiment performed in this study can be found using the accession number GSE166458 (CEL datasets).
https://www.ncbi.nlm.nih.gov/geo/query/acc.cgi?acc=GSE166458
The secure token for reviewers: ohupuugsbzgbpor
Original data relating to the the microarray experiment can also be accessed from the Stowers Original Data Repository
https://www.stowers.org/research/publications/LIBPB-1647
The raw data associated with in vitro and in vivo RNAseq analysis used in this study can be found using the accession number GSE180427.
https://www.ncbi.nlm.nih.gov/geo/query/acc.cgi?acc=GSE180427
The secure token for reviewers: idqtogyarlidhmp

# Field-specific reporting

Please select the one below that is the best fit for your research. If you are not sure, read the appropriate sections before making your selection.

- [x] Life sciences
- [ ] Behavioural & social sciences
- [ ] Ecological, evolutionary & environmental sciences

For a reference copy of the document with all sections, see nature.com/documents/nr-reporting-summary-flat.pdf

# Life sciences study design

All studies must disclose on these points even when the disclosure is negative.

| | |
|---|---|
| Sample size | For qualitative determination of gene expression differences using whole-mount in situ hybridisation, a minimum of 2 biologically distinct samples per time-point and per genotype were assessed.<br>For quantitative determination of gene expression using Fluidigm (Figure 1), a minimum of 3 biological samples per genotype and per time-point were assessed.<br>For quantitative determination skeletal number and morphology, a minimum of 2 and maximum of 14 biological distinct samples were used per genotype<br>For quantitative determination of cell populations by flow cytometry, a minimum of 4 and maximum of 7 biological samples per genotype were assessed. |
| Data exclusions | No data was excluded. |
| Replication | Each embryo/skeleton is considered an individual sample, and thus replicate number = sample size. The reproducibility for each experiment is detailed in the main text or supplementary figures. |
| Randomization | Allocation of experimental groups was set out by mouse genotypes. |
| Blinding | All skeletons were scored for vertebral number and morphology by 2 researchers, blinded to genotype. Any instance where there was discrepancy in result, the skeleton in question was scored by a third individual, also blinded to genotype. |

# Reporting for specific materials, systems and methods

We require information from authors about some types of materials, experimental systems and methods used in many studies. Here, indicate whether each material, system or method listed is relevant to your study. If you are not sure if a list item applies to your research, read the appropriate section before selecting a response.

## Materials & experimental systems

| n/a | Involved in the study |
| --- | --- |
| ☐ | ☒ Antibodies |
| ☐ | ☒ Eukaryotic cell lines |
| ☒ | ☐ Palaeontology and archaeology |
| ☐ | ☒ Animals and other organisms |
| ☒ | ☐ Human research participants |
| ☒ | ☐ Clinical data |
| ☒ | ☐ Dual use research of concern |

## Methods

| n/a | Involved in the study |
| --- | --- |
| ☒ | ☐ ChIP-seq |
| ☒ | ☐ Flow cytometry |
| ☒ | ☐ MRI-based neuroimaging |

# Antibodies

| | |
| --- | --- |
| Antibodies used | Anti-Digoxigenin Fab fragments Antibody, AP Conjugated, Roche, Cat# 11093274910, RRID:AB_514497<br><br>Validation of In vitro differentiation kinetics is routinely performed in lab (not described in detail in main text but referred to in Methods section) using the following antibodies:<br>Primary antibodies: rabbit anti-T/Brachyury (T) antibody (AbCam, Ab209665) and rat anti-SOX2 antibody (AbCam, Ab92494).<br>Secondary antibodies: anti-rat AlexaFluor 488 (ThermoFisher, A212208) and anti-rabbit AlexaFluor 555 (ThermoFisher, A31572).<br><br>Flow cytometry analysis of tailbud cell populations used the following antibodies:<br>Primary: Rat anti-Sox2 (Btjce, eBiosciences, 14-9811-82); Rabbit anti-T/Brachyury (EPR18113, Abcam, ab209665).<br>Secondary: Goat anti-rat IgG, AF555 (Invitrogen, A48263); Goat anti-rabbit IgG, AF647 (nvitrogen, A32733). |
| Validation | All primary antibodies have been used in numerous previously published studies on the cell types investigated. However, antibody lots can be variable so each antibody was verified in house to replicate the correct expression pattern and cell location within the tissues tested. Additionally, all primary and secondary antibodies have been tested to ensure there was no non-specific binding. |

# Eukaryotic cell lines

Policy information about cell lines

| | |
| --- | --- |
| Cell line source(s) | Wildtype Bruce4 mouse embryonic stem cell (ESC) line isolated from C57BL/6 mouse strain and an in-house generated Nr6a1 knockout line derived from the same ESC line. |
| Authentication | The Wildtype ESC line has been published: Abbondanzo et al. (1993) PMID 8231888. Multiple independent Nr6a1 knockout lines were produced, sequence verified and tested for their ability to generate NMPs, with data presented for the clone used in these studies (Supp Figures 8 and 9). |
| Mycoplasma contamination | All ESCs and mouse embryonic fibroblast for feeder layers were routinely tested and confirmed mycoplasma free. |
| Commonly misidentified lines (See ICLAC register) | Only wildtype ESC and one in-house generated modified ESC were used. |

# Animals and other organisms

Policy information about studies involving animals; ARRIVE guidelines recommended for reporting animal research

| | |
| --- | --- |
| Laboratory animals | Wildtype (C57BL6), Gdf11-KO, miR196-TKO, CMV-CreERT2, T-CreERT2, Nr6a1flx/flx, Cdx2P:Nr6a1. Embryonic stages assessed are described in the text for each figure. |
| Wild animals | No wild animals were used in this study. |
| Field-collected samples | No field-collected samples were used in this study. |
| Ethics oversight | All animal procedures performed at Monash University were performed in accordance with the Australian Code of Practice for the Care and Use of Animals for Scientific Purposes (2013). Those experiments were approved by the Monash Animal Ethics Committee under project number 21616. Animal experiments performed at the Stowers Institute for Medical Research were conducted in accordance with the Institutional Animal Care and Use Committee approved protocol IACUC #2019-097. |

Note that full information on the approval of the study protocol must also be provided in the manuscript.
